# From antagonism to synergism: Extreme differences in stressor interactions in one species

**DOI:** 10.1038/s41598-020-61371-x

**Published:** 2020-03-13

**Authors:** Lars Straub, Angela Minnameyer, Verena Strobl, Eleonora Kolari, Andrea Friedli, Isabelle Kalbermatten, Antoine Joseph Willem Marie Merkelbach, Orlando Victor Yañez, Peter Neumann

**Affiliations:** 1 Institute of Bee Health, Vetsuisse Faculty, University of Bern, Bern, Switzerland; 2 Agroscope, Swiss Bee Research Centre, Bern, Switzerland

**Keywords:** Environmental impact, Environmental impact, Behavioural ecology, Conservation biology

## Abstract

Interactions between stressors are involved in the decline of wild species and losses of managed ones. Those interactions are often assumed to be synergistic, and *per se* of the same nature, even though susceptibility can vary within a single species. However, empirical measures of interaction effects across levels of susceptibility remain scarce. Here, we show clear evidence for extreme differences in stressor interactions ranging from antagonism to synergism within honeybees, *Apis mellifera*. While female honeybee workers exposed to both malnutrition and the pathogen *Nosema ceranae* showed synergistic interactions and increased stress, male drones showed antagonistic interactions and decreased stress. Most likely sex and division of labour in the social insects underlie these findings. It appears inevitable to empirically test the actual nature of stressor interactions across a range of susceptibility factors within a single species, before drawing general conclusions.

## Introduction

In light of the recent reported high losses of biodiversity within the past centuries^1,2^, it is apparent that the Earth is undergoing its sixth mass extinction event^3,4^. While the often charismatic megafauna has long been the focus, insects have only recently received attention^5^, despite their indispensable role for both functionalities of terrestrial ecosystems and human food security^6,7^. Indeed, mounting evidence revealing both global declines in insect biomass as well as the diversity of insect pollinators has raised great concern^8–10^. A wide array of drivers are held responsible for the reported declines, including global climate change, habitat loss, intensified agricultural practices as well as the spread of pests and pathogens^11–13^.

Naturally, these stress factors act upon our environment simultaneously^14^, causing complex interactions that may mitigate or exacerbate effects on an individual species or population^15^. The potential negative consequences of such interactions upon wild insect populations have been shown in both North America and Britain, where intensified agriculture in combination with the loss of nutritional resources or diseases caused severe declines in pollinator species^16,17^. Subsequently, there is a general consensus that the interactions of combined stressors are a highly plausible explanation for recent species extinctions and population declines. However, a vast knowledge gap remains in understanding how susceptibility may vary amongst species facing combined stressor scenarios.

Inter- and intra-specific species variability in stressor sensitivity is known^18^. For instance, biotic homogenization is likely to impose larger consequences on specialist insects compared to generalists^13^. Thus, extrapolating stressor effects from one species to another without considering fundamental differences in life-history traits or phenology may not be appropriate^19^. Furthermore, within a species, differences in age groups^20^, developmental stages^21^ and sex^22,23^ may play a crucial role in understanding susceptibility. This is further underlined by the importance of considering varying genetics as a key factor^24^. Moreover, in various insect orders (Hymenoptera, Thysanoptera, and Coleoptera), the haplo-diploid sex determination system, where females are diploid and males usually develop from unfertilized eggs and are haploid, reveals an additional level of intricacy^25^. This becomes evident when considering the haploid-susceptibility hypothesis, which postures that a lack of heterozygosity at immune loci may result in reduced immunocompetence^26^, yet empirical data remain scarce. Lastly, the possible influence of reproductive division of labour, one cornerstone of the biology of social insects^27^, remains relatively unexplored^28^, despite colony demographics and polyethism having been shown to influence disease susceptibility^29^.

The eusocial western honeybee, *Apis mellifera*, has historically served as a model organism to investigate the effects of environmental and anthropogenic stressors, mainly due to its role as a managed pollinator species, as well as their comparatively well-studied biology^30,31^. By taking advantage of division of labour and complementary sex determination^32^ in the honeybees, we aim to test possible different levels of susceptibility in haploid male (drone) and diploid female (worker) bees towards two common honeybee stressors: an obligatory intracellular midgut parasite, *Nosema ceranae*, and malnutrition. Both *N. ceranae* and poor nutrition can compromise immunocompetence^33,34^ and individual bee physiology (e.g. reduced body mass^35,36^) which may ultimately explain increased mortality rates^37^. Considering previous studies and the expectations from the haploid-susceptibility hypothesis, we hypothesize that the combined treatments will not only reveal significant negative synergistic effects upon individuals, but that these effects will be amplified in the haploid drones.

## Results

### Consumption

No significant differences were found for sugar water consumption amongst treatment groups (${F}_{3,270}$ = 2.4, *P* > 0.05; Electronic Supplementary Material ESM Figure S2A), with the average daily bee consumption ranging between 38.11 ± 11.08 mg and 40.72 ± 8.62 mg (mean ± S.D.; ESM Table S3). Median pollen consumption for Controls (2.08 ± 0.34 − 6.24 mg) did not differ from Pathogen (2.48 ± 0.30 - 7.43 mg) (${F}_{1,136}$ = 0.60; *P* > 0.05; median 95% C.I.; ESM Table S3). The average daily pollen consumption ranged between 1.85 ± 1.61 mg and 2.63 ± 2.12 mg (mean ± S.D; ESM Table 2). Pollen consumption significantly differed over the experimental period (${F}_{5,125}$ = 104.88; *P* < 0.05; ESM Figure S2B).

### Survival

#### Drones

**Figure 1:**
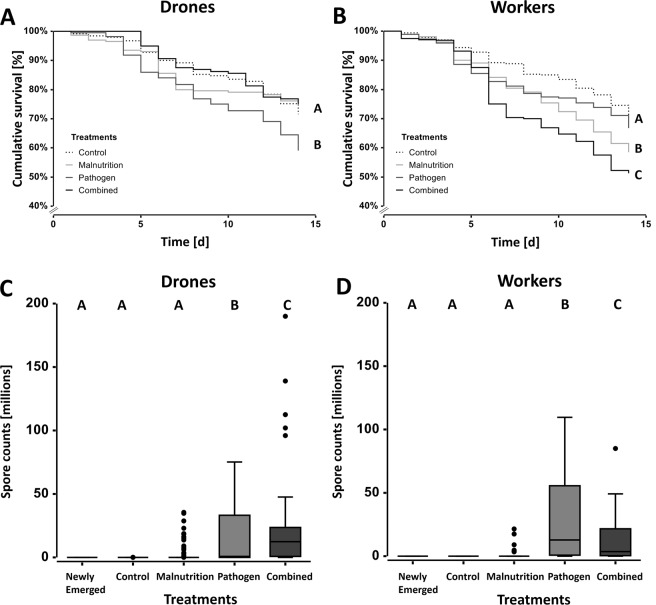
Honeybee drone and worker cage mortality and *Nosema ceranae* spore loads. (**A, B**) Survival curves (Kaplan-Meier) indicate the cumulative survival [%] of honeybees over the 14-day experiment for each treatment. In drones, the Pathogen treatment had significantly lower survival when compared to the remaining treatments. In workers, the Combined treatment had the lowest survival, and the Malnutrition treatment was significantly lower than the Control and Pathogen. Different letters indicate a significant difference between treatments. **(C,  D)** *N. ceranae* spore loads of individual honeybee drones and workers for each treatment group. For drones, the Pathogen and Combined had significantly higher spore counts than the remaining treatments, but did not differ themselves. For workers, Pathogen had significantly higher spore counts than all other treatments. The boxplots show the inter-quartile range (box), the median (line within box), data range (horizontal lines from box), and outliers (black dots). Different letters indicate a significant difference between treatments.

Median cumulative survival [%] at day 14 for Malnutrition (76.1 ± 70.6 - 81.6) and Combined (76.8 ± 70.3 - 83.4) treatments did not significantly differ from Controls (75.2 ± 69.9 - 80.6) (all *Ps* > 0.483, median ± 95% C.I.; Fig. 1A). In contrast, Pathogen (64.5 ± 58.2 - 70.6) had significantly lower survival when compared to Controls and the remaining treatments (all *Ps* < 0.003, median ± 95% C.I.; Fig. 1A), which resulted in a reduction in survival of 14%. The Combined treatment lead to an antagonistic interaction and showed decreased stress when compared to their relative single stressor treatments (ESM Table S4).

#### Workers

No significant difference in median cumulative survival [%] was observed between Controls (74.6 ± 70.8 - 78.42) and Pathogen (71.1 ± 66.9 - 75.37) (*P* = 0.102). In contrast, Malnutrition (61.52 ± 57.08 - 65.97) and Combined (52.2 ± 46.7 - 57.7) resulted in significant reductions of survival (all *Ps* < 0.001, median ± 95% C.I., Fig. 1B), whereby survival was reduced by 18% and 30%, respectively. The Combined treatment revealed a synergistic interaction and increased stress compared to the single stressors (ESM Table S4).

### Body mass

#### Drones

All treatment groups revealed significant reductions in body mass (9% - 16%) 14 days post-emergence when compared to the Newly Emerged drones (261.2 ± 23.6) (all *Ps* < 0.001; mean ± S.D.; ESM Figure S3A). Pollen fed bees from the Pathogen treatment (235.6 ± 25.3 mg) did not significantly differ from the Controls (239.0 ± 24.0 mg) (*P* = 1.00; mean ± SD; ESM Figure S3A). In contrast, treatment groups without pollen showed significantly reduced body mass compared to Controls (all *Ps* < 0.001, ESM Figure S3A). This translated to a reduction in body mass for the Malnutrition (218.5 ± 24.24 mg) and Combined (220.7 ± 19.9 mg) of 8.6%, and 7.6%, respectively (mean ± 95% C.I.). The Combined treatment resulted in an antagonistic interaction and showed decreased stress when compared to their respective single stressor treatments (ESM Table S4).

#### Workers

Individuals from the treatment groups deficient of pollen did not significantly differ from the Newly Emerged workers (107.6 ± 12.5 mg) 14 days post-treatment initiation (all *P*s > 0.585; ESM Figure S3B). In contrast, individuals from treatment groups that were fed a pollen diet showed a significant increase in body mass post-treatment initiation (all *Ps* < 0.001; ESM Figure S3B). Control (135.3 ± 15.5 mg) and Pathogen (135.8 ± 14.3 mg) revealed the highest increase in body mass (~26%) and were significantly heavier than Combined individuals (107.9 ± 21.8) (all *Ps* < 0.001; mean ± S.D.; ESM Figure S3B). The Combined treatment lead to a synergistic interaction and showed increased stress when compared to their respective single stressor treatments (ESM Table S4).

### *Nosema ceranae* spore counts

#### Drones

No *N. ceranae* spores were detected in the Newly Emerged individuals; however, spores were detected in both Control (0 ± 0–0 million) and Malnutrition (0 ± 0–18.8 million) (median ± 95% C.I.; Fig. 1C). Nevertheless, Control and Malnutrition did not significantly differ from the Newly Emerged treatment group (all *Ps* > 0.885, median ± 95% C.I.; Fig. 1C). The Pathogen (0.025 ± 0–65.3 million) and Combined (0 ± 0–102 million) treatment groups showed a significant increase in spores when compared to Controls (all *P*s < 0.001), yet they did not significantly differ from one another (*P* = 0.592, median ± 95% C.I., Fig. 1C). The Combined treatment resulted in an antagonistic interaction and decreased stress when compared to their respective single stressor treatments (ESM Table S4).

#### Workers

*N. ceranae* spores were not detected in Newly Emerged or Control treatment groups, whereas spores were found in all other treatment groups. Despite *N. ceranae* spore detection, Malnutrition (0 ± 0–9 million) and Combined (0 ± 0.0 - 41.3 million) did not significantly differ when compared to Controls (0 ± 0–0 million) (all *Ps* > 0.113); median ± C.I.; Fig. 1D). Significantly increased *N. ceranae* spore counts were detected in the Pathogen treatment (0.6 ± 0 - 81.8 million) when compared to all other treatments (all *Ps* < 0.001). This subsequently resulted in an antagonistic interaction and decreased stress for the Combined treatment when compared to their respective single stressor treatments (ESM Table S4).

### Comparison between drones and workers

#### Consumption

Comparisons between the consumption rates of drones and workers were not possible due to the design of the experiment, whereby both were maintained within the same cage.

#### Survival

**Figure 2:**
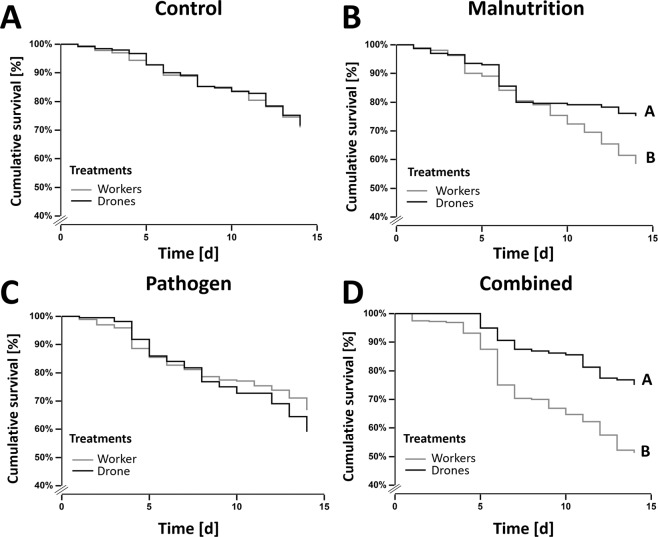
Honeybee drone and worker cage mortality. Survival curves (Kaplan-Meier) compare the cumulative survival [%] of honeybee (*Apis mellifera*) workers (grey line) and drones (black line) over the 14-day experiment for each individual treatment: (**A**) Control, (**B**) Malnutrition, (**C**) Pathogen, (**D**) Combined. The data revealed that workers and drones receiving pollen (**A & C**) did not significantly differ from one another, whereas workers deprived of pollen (**B & D**) showed significantly lower survival rates then pollen deprived drones.

Median longevity did not significantly differ between drones and workers for Control or Pathogen treatment groups (both *Ps* > 0.087; Fig. 2A,C). In sharp contrast, the non-pollen treatments (Malnutrition and Combined) consistently revealed that workers showed significantly reduced survival rates when compared to drones (all *Ps* < 0.001; Fig. 2B,D), resulting in reduced median longevity by 14.6% and 24.7%, respectively. Drones from the Pathogen treatment revealed the lowest median longevity, whereas the workers from the Combined treatment revealed the lowest median longevity, subsequently leading to contrasting interaction effects between drones (antagonistic) and workers (synergistic) (ESM Table S4).

#### Relative body mass

A clear sex difference was observed for body mass 14 days post-treatment initiation. When compared to Newly Emerged individuals, drones revealed significantly reduced body mass (all *Ps* < 0.001), whereas workers either did not significantly differ or significantly increased. Relative to Controls, body mass loss was greater in workers than in drones for both Malnutrition and Combined treatments, with workers showing increased reductions of 7.27% and 12.62%, respectively. Additionally, contrasting interaction effects were found between drones (antagonistic) and workers (synergistic) (ESM Table S4).

#### *Nosema ceranae* spore counts

No significant differences in infection rates between drones and workers were found (all *Ps* > 0.067). Regardless of the treatment group, no significant differences in median spore counts were observed between drones and workers (all *Ps* > 0.166). No significant correlations between body mass and *N. ceranae* were found for drones (Pearson correlation |r_342_ | = −0.038, df = 340, *P* = 0.485) or workers (Pearson correlation |r_164_ | = 0.0326, df = 162, *P* = 0.687). Both drones and workers revealed the same interaction effects (antagonistic and reduced stress; ESM Table S4). No significant difference in infection efficiency was observed between workers and drones for all treatment groups (all *P*s > 0.067). Likewise, no difference in infection efficiency was observed when comparing Pathogen to Combined (χ² = 2.45, DF = 1, *P* = 0.118).

## Discussion

Our data show distinct stressor interactions within a single species. While diploid female honeybee workers exposed to both malnutrition and the pathogen *N. ceranae* showed synergistic interactions and increased stress, haploid male drones showed antagonistic interactions and decreased stress. Division of labour in the social insects apparently overrides any possible disadvantage of hemizygosity as predicted by the haploid-susceptibility hypothesis. Our study emphasizes the urgent need to empirically test the actual nature of stressor interactions across a range of susceptibility factors within a model system.

Our findings must be interpreted within the context of laboratory conditions and definite methodological differences to other studies (i.e. bulk vs. hand feeding, varied spore solutions). Indeed, our cage set-up greatly improved drone survival under laboratory conditions, which is historically low^38,39^. By limiting the extreme stress of cages on drones, we achieved the same survival rates in drones and workers in the Controls, allowing for a direct comparison of treatment effects between the honeybee sexes. Since the pollen was not irradiated, this explains the *N. ceranae* infections in the Controls^37,40^. Nevertheless, spore counts did not significantly differ amongst non-pathogen-exposed groups, including the newly emerged individuals, subsequently having no significant effect. We found no significant differences in sucrose consumption between treatments, which is in line with previous studies^40,41^. Since other studies found effects^37,42,43^, it is evident that infection with *N. ceranae* does not necessarily lead to increased hunger levels. Our data also show no difference amongst treatments for pollen consumption^37,44^. However, pollen consumption was highest during the first week when newly emerged bees utilize protein for organ and tissue development^40,45^. Indeed, the Malnutrition treatment reduced body mass in both drones (9%) and workers (16%) compared to their relative Controls (as in^37^), which may have an impact on bee performance^46^.

While malnutrition alone caused significant worker mortality (18%), in line with previous studies^37,40,47^, this was not the case in drones. Since workers have higher pollen requirements compared to drones^48^ due to division of labour (e.g. jelly production^49^), it appears evident that lack of protein will cause more stress in the workers. Furthermore, feeding other bees is costly and can reduce worker lifespan^50^. Therefore, the attending nurse bees in our experiment were *per se* more active than the drones, who in sharp contrast received attendance.

No difference in infection rates, spore loads or survival were found between the Pathogen treatment drones and workers. Nevertheless, our data confirm that *N. ceranae* infected workers (Pathogen treatment) display higher spore loads when pollen-fed^37,43^. The addition of pollen, however, did not impact drone spore load, again possibly due to their more limited intake of pollen^49^. However, drones from the Pathogen treatment had a lower survival compared to their Control and Combined treatments, which was not the case for workers. Since spore loads did not differ between Combined and Pathogen treatments in drones, this suggests that higher mortality in the Pathogen treatment is not induced by *N. ceranae* itself. Alternatively, starvation due to less efficient attending of highly infected workers in the Pathogen treatment may explain this phenomenon^43^.

The data from the Combined treatments were most remarkable. In sharp contrast to our predictions based on the published literature, stressor interactions were antagonistic in drones and synergistic in workers. Therefore, the data do not support the haploid-susceptibility hypothesis^51,52^, predicting that effects should be amplified in the haploid drones. Indeed, drones showed antagonistic effects and decreased stress, wherein the Combined treatment survival, surprisingly, did not differ from the Controls. On the other hand, worker exposure to combined stressors revealed synergistic effects and increased stress. A significantly reduced worker survival and relative body mass was found compared to both Controls and drones from their respective treatment. Since the Combined exposed workers revealed the lowest survival of all groups, yet had fewer spores than the Pathogen treatment (confirming^37^), this clearly shows the synergistic and increased stress effect. This highlights the importance of adequate protein nutrition for worker tolerance to pathogen infections^53^ and attending nest mates^48^. Indeed, workers are actually exposed to three and not only two stressors. Besides lack of protein and the pathogen, workers are confronted with social stress imposed by male nest mates actively seeking attention, especially within the first few days of emergence^54^. It therefore appears evident that the stressor interactions are different between the sexes. In general, *nomen est omen*, hence workers are performing all tasks to maintain a functional colony due to division of labour in the social insects^27^. Therefore, life-history differences between drones and workers may outweigh the potential negative effect of hemizygoisity at loci towards these stressors. Workers are usually short-lived, replaceable units that do not normally reproduce^27^. On the other hand, drones are sexuals and their survival is essential for reproduction and colony fitness^55^. Therefore, superorganism resilience, the ability to tolerate the loss of somatic cells (=workers) as long as the germline (=reproduction) is maintained^56^, may ultimately explain why drones are actually performing better than workers. Workers can be replaced easily and a high turnover rate may even be adaptive at the colony level, e.g. not enabling ample pathogen reproduction^56,57^.

Division of labour in the social insects is just one factor driving susceptibility to stressor interactions in a species. Other drivers are likely to be ontogenetic^58^, senescence^59^, sex and polymorphism (e.g. winter vs summer honey bees^60^). In this particular case, the workers were the weakest link. It is apparent that this may be very different in other cases (e.g. in case of drones and pesticides^61^). Therefore, we suggest an *a priori* screening of the model system for the chances of the susceptibility factors to occur and the actual impact they have at individual and population level. In light of the documented importance of stressor interactions^11–13^, it appears prudent to take those points into account to ensure efficient nature conservation efforts and sustainable food security.

## Conclusion

Our study provides clear evidence for extreme differences in stressor interactions within a single species, ranging from antagonism to synergism. It, therefore, appears inevitable to consider a range of factors known to govern the susceptibility towards stressor interactions such as ontogenetic, senescence, sexes and division of labour in the social insects, as shown here. Most importantly, multiple stressor interactions cannot be regarded as synergistic *per se*, but need to be empirically tested across a range of possible susceptibility factors.

## Material and methods

### Experimental design

The experiment was conducted in June and July 2018 at the Institute of Bee Health, University of Bern, Switzerland, using seven local, non-related and queenright *A. mellifera* colonies and Best Management Practices, incl. an oxalic (2.7%) acid *Varroa destructor* treatment in the previous winter and early-spring^62^.

### Source of drones and workers

To obtain sufficient drones and workers of a known age, all queens were caged in their colonies for 48 hours on frames with organic drone and worker wax foundations. Brood frames were transferred 24 hours prior to adult emergence to a laboratory incubator maintained at 34.5 °C and 60% RH in darkness^63^. To foster drone emergence and feeding, ~50 adult worker from each colony were added to their respective drone frame^64^. Post-emergence, drones and workers without clinical symptoms of disease^65–67^ were randomly placed in standard hoarding cages [250 cm^3^]^68^.

### *Nosema ceranae* cultivation and inoculation

Spore solutions were freshly obtained using routine protocols, including tests with species-specific PCR primers^69^. Five *N. ceranae* positive foragers (all 20 negative for *Nosema apis* (ESM Figure S1 and Table S1) were used to infect newly emerged, caged workers via bulk feeding^70^ to obtain spore solutions of known concentrations^35,71^.

### Treatments

To investigate sub-lethal and lethal effects of malnutrition and *N. ceranae* infections, singly and in combination, on drones and workers, a fully-crossed hoarding cage experiment was designed using the following four feeding treatment groups: 1. Sucrose solution and pollen (=Controls), 2. Sucrose solution only (=Malnutrition), 3. Sucrose solution, pollen, plus ~10,000 *N. ceranae* spores/bee (=Pathogen) and, 4. Sucrose solution only, plus ~10,000 *N. ceranae* spores/bee (=Combined) (ESM Table S2). All bees were starved for two hours^37,72^ before solutions were provided via bulk feeding^70^. Optimal nutritional conditions were provided to Control and Pathogen treatment groups by enabling access to both a carbohydrate (sucrose solution) and protein source (corbicular pollen)^36^. In contrast, the Malnutrition and Combined treatment groups lacked a protein source, thereby imitating nutritional stress. The provided corbicular pollen was not gamma-ray irradiated. Bulk feeding of *N. ceranae* occurred only within the first 24 hours of the experiment. Once the spore suspension had been consumed, it was replaced with a pathogen-free 50% [w/w] sucrose solution in all cases.

### Hoarding cages

In total, 96 hoarding cages (22–26 per treatment group) were each filled with 10 drones and 20 workers^68^ (ESM Table S2), randomly assigned to a treatment group and were maintained in complete darkness at 30 °C and 60% RH^63^. All cages contained a 5 ml syringe providing 50% [w/v] sucrose solution *ad libitum* to provide sufficient carbohydrates. Depending on the treatment group, hoarding cages contained an additional 2.5 ml Eppendorf feeder providing *ad libitum* pollen paste (70% fresh corbicular pollen, 30% powder sugar) as a protein source^36^.

### Food consumption and mortality

Sucrose solution and pollen paste consumption were weighed every other day to test for differences in nutritional demand^42^. The sugar water syringes were replaced after being weighed to avoid potential mold formation^63^. Since the average weight loss of sucrose solution from the syringes due to evaporation (<1%) was negligible (three empty cages kept in the incubator), this factor was excluded. The daily sugar consumption per bee [mg] was calculated by correcting for the number of individuals alive per cage over the 48-hour time period^73^. We tested the sugar consumption in 12 random cages per treatment group (N = 48) at six time points throughout the experiment (N = 271). Pollen consumption was calculated in the same way, however, only the treatments fed with pollen were used (N = 24 cages, 137 measurements). Mortality was recorded daily, whereby dead bees were counted and removed. Cages with non-functional feeders (N = 10) were excluded. Both consumption and survival were monitored until the experiment was terminated 14 days post-treatment initiation^39,70,74^. In total, we monitored the survival of 2,880 bees.

### Bee body mass and *Nosema ceranae* spore counts

Teneral body mass (drones: N = 238, workers: N = 240) and *N. ceranae* spore counts (drones and workers: N = 80 each) were determined for individual drones and workers upon emergence and 14 days post-treatment (drones: N = 360, workers: N = 210)^35^.

### Statistical analyses

All tests and figures were performed using NCSS 2019^75^. Data were tested for normality using the Shapiro-Wilk’s test and visually inspected using Q-Q-plots^76^. While body mass and sucrose consumption were normally distributed (Shapiro-Wilk’s test, *P* > 0.05, ESM Table S3) and analysed using a One-way ANOVA, pollen consumption per bee and *N. ceranae* spore counts were non-normally distributed (Shapiro- Wilk’s test, *P* < 0.05, ESM Table S3) and analysed using a Kruskal-Wallis One-way ANOVA^76^. Post-hoc comparisons of all variables were conducted by using a multiple pairwise comparisons test (Bonferroni Multiple Comparison Test (bmct)). Additionally, pollen consumption per bee over time was evaluated using repeated measures ANOVA (Mauchly’s Test for Sphericity was not significant (*P* > 1.00)). Survival analyses were performed using Kaplan-Meier cumulative survival curves and Log-Rank values were calculated to determine differences amongst treatment groups. An XY scatter plot and the Pearson’s correlation coefficient was used to assess for a potential correlation between body mass and *N. ceranae* spore counts. Additionally, χ^2^-tests were used to compare infection rates between treatments and between drones and workers.

### Interactions

To investigate interaction effects between malnutrition and *N. ceranae* we employed an additive effects model^77,78^. In the additive model, synergism or antagonism occur when the combined effect of multiple stressors is greater (synergism) or less (antagonism) than the sum of effects elicited by individual stressors^79^. Additionally, to gain clarification on the degree of stress, the simple comparative model was also applied^77^. This model states that increased or decreased stress occurs when the combined effect of multiple stressors is greater (increased) or less (decreased) than the effect of the single worst stressor. Interactive stress effects on consumption, body mass and survival were calculated as the percent differences in treatments relative to controls, whereby the mean body mass [mg], median cumulative survival [%] and median *N. ceranae* spore counts [spores bee^−1^ millions] at day 14 were used for the calculations (ESM Table S4).

## Supplementary information

Supplementary Material.

## Data Availability

The complete raw data can be found at the Dryad repository. 10.5061/dryad.v9s4mw6r7
