# Supplementary material for: From antagonism to synergism: Extreme differences in stressor interactions in one species
Source: Sci Rep. 2020 Mar 13;10:4667. doi: 10.1038/s41598-020-61371-x (PMC7069998; doi:10.1038/s41598-020-61371-x)
Supplement: Supplementary file 1 — Supplementary Material. [file 41598_2020_61371_MOESM1_ESM.docx]

**Electronic supplementary material
Title:** From antagonism to synergism: Extreme differences in stressor interactions in one species

**Authors and affiliations:**

Authors: Lars Straub*†^1,2^, Angela Minnameyer*†^1^, Verena Strobl^1^, Eleonora Kolari^1^, Andrea Friedli^1^, Isabelle Kalbermatten^1^, Antoine Joseph Willem Marie Merkelbach^1^, Orlando Victor Yañez^1^, Peter Neumann^1,2^

1Institute of Bee Health, Vetsuisse Faculty, University of Bern, Bern, Switzerland.

2Agroscope, Swiss Bee Research Centre, Bern, Switzerland.

† Contributed equally

* Corresponding Authors:
[lars.straub@vetsuisse.unibe.ch](mailto:lars.straub@vetsuisse.unibe.ch)

[angela.minnameyer@vetsuisse.unibe.ch](mailto:angela.minnameyer@vetsuisse.unibe.ch)

**Abstract**

Interactions between stressors are involved in the decline of wild species and losses of managed ones. Those interactions are often assumed to be synergistic, and *per se* of the same nature, even though susceptibility can vary within a single species. However, empirical measures of interaction effects across levels of susceptibility remain scarce. Here, we show clear evidence for extreme differences in stressor interactions ranging from antagonism to synergism within honeybees, *Apis mellifera*. While female honeybee workers exposed to both malnutrition and the pathogen *Nosema ceranae* showed synergistic interactions and increased stress, male drones showed antagonistic interactions and decreased stress. Most likely sex and division of labour in the social insects underlie these findings. It appears inevitable to empirically test the actual nature of stressor interactions across a range of susceptibility factors within a single species, before drawing general conclusions.

**Keywords:** honeybees, pathogens, malnutrition, stress interactions, haploid-susceptibility, drones


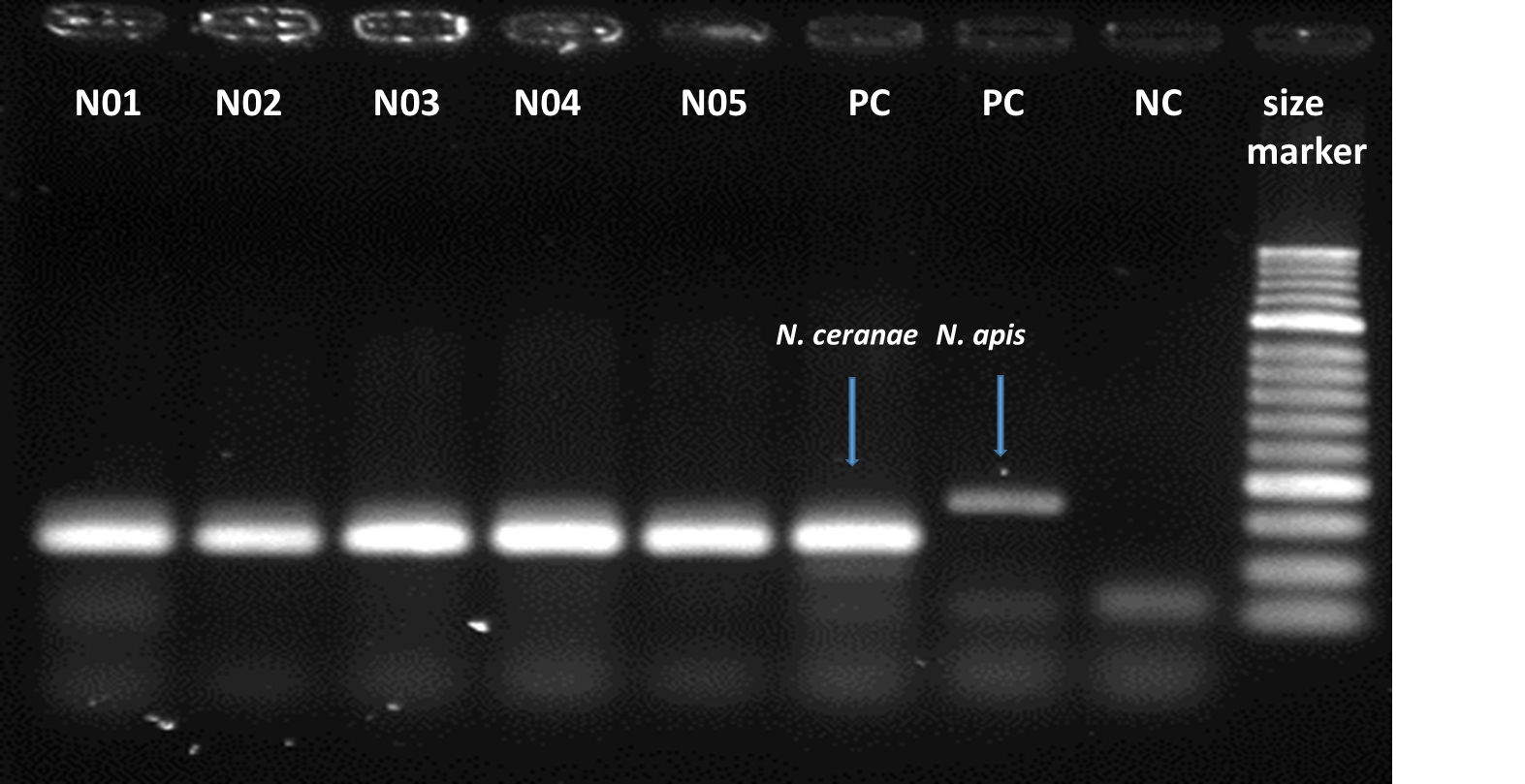


**Figure S1 | Gel electrophoresis:** Positive results of the PCR (N0X = individual bee, PC = positive control, NC = negative control, size marker = molecular-weight size marker, also referred to as a DNA ladder).

**Table S1 | Primer sequence:** Forward and reverse primer sequences used in the PCR to identify *Nosema ceranae* and *Nosema apis*.


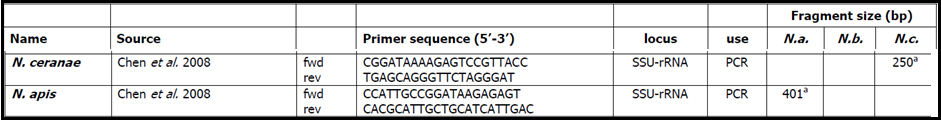


**Table S2** **| Overview of experimental treatment groups, stressors and sample sizes used for the cage experiments:** Nutrition, stressors, and sample size (N) information for each treatment group is reported. *Nosema ceranae* spores were fed to the bees in the pathogen exposed treatments (10'000 spores bee^-1^) via sugar feeders. Bees deprived of pollen were either in the Malnutrition or Combined treatment group. Stressor level (single or combined) was used to calculate interaction effects between the exposed stressors. Presence of either sugar water and pollen or a specific stressor is indicated by a positive symbol (**+)**.

Table S3 | Summary of statistical results obtained for the effects of malnutrition, pathogen and combined exposure on honeybee, Apis mellifera (L.), worker and drones for all measured variables.

**Table S4 | Summary of interactions between malnutrition and *Nosema ceranae* on honeybee (*Apis mellifera*) workers and drones for all outcome variables.** Based upon the additive effects and simple comparative effects model, stressors effects were calculated as the percent difference in treatments relative to controls, whereby the median longevity [d], body mass [mg] *and N. ceranae* spores per bee [millions] were used for the calculations. Synergistic and antagonistic effects were determined by comparing the sum of individual effects with the effects of the combined stressors. Synergism or antagonism occurs when the combined effect of multiple stressors is greater (synergism) or less (antagonism) than the sum of effects elicited by individual stressors. The degree of stress was assessed using the comparative model, whereby increased or decreased stress occurs when the combined effect of multiple stressors is greater (increased) or less (decreased) than the effect of the single worst stressor.

**
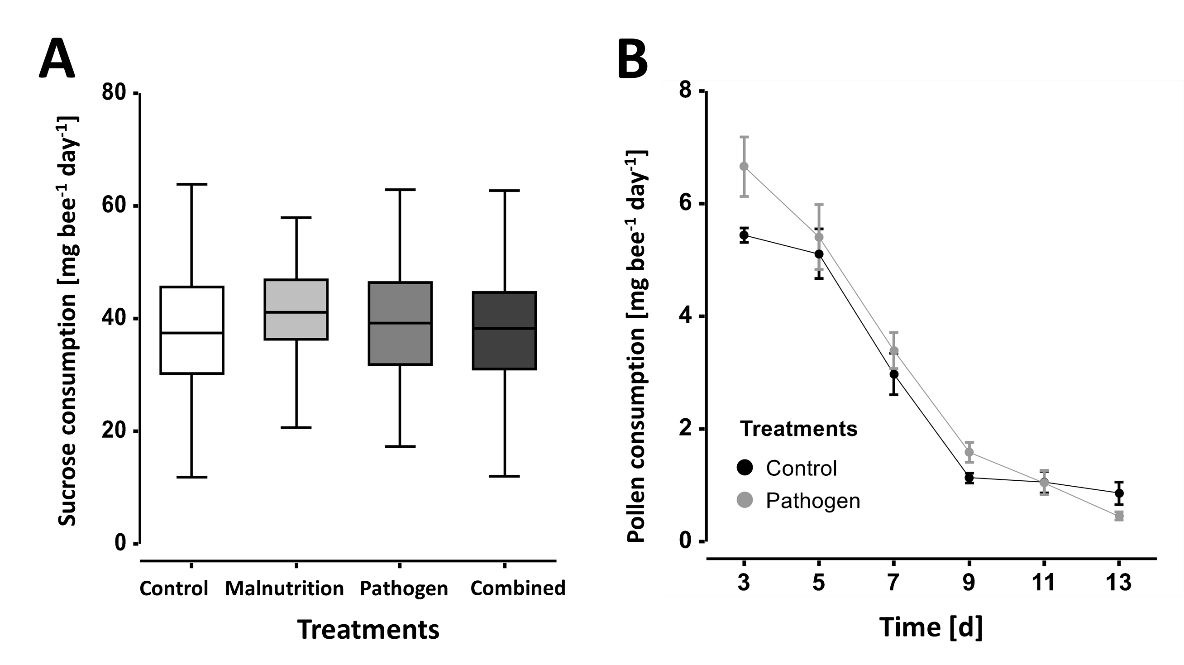
 Figure S2 | Daily sucrose and pollen consumption over 14 days. A)** Sucrose consumption [mg bee⁻¹]. No significant difference was found between the treatments (*P*-value > 0.05). The boxplots show the inter-quartile range (box), the median (black line within box), data range (horizontal black lines from box), and outliers (grey dots). **B)** Pollen consumption [mg bee⁻¹] over time. There were no significant differences between the treatments.


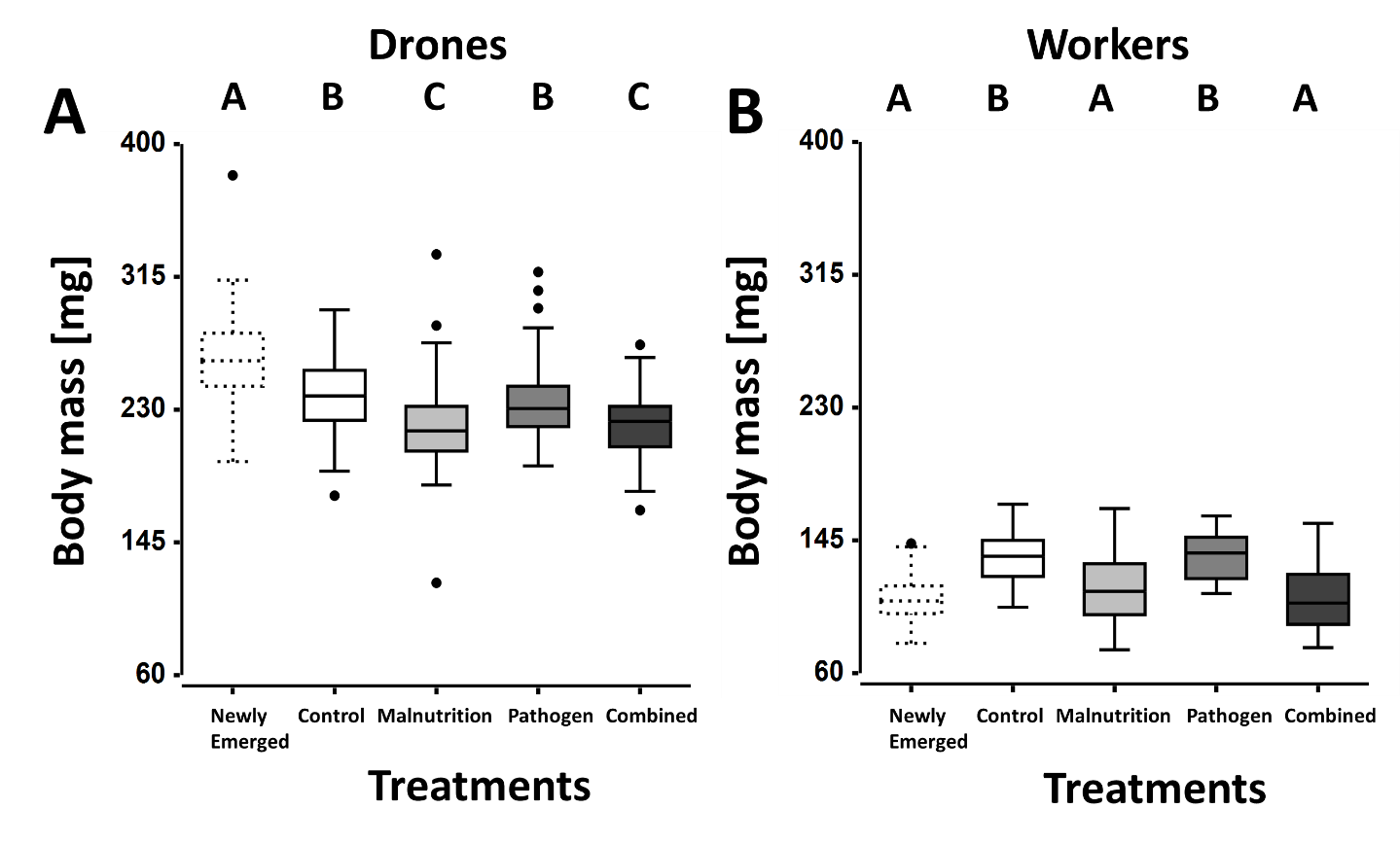


**Figure S3 | Honeybee drone and worker body mass.** Body mass [mg] reported across treatments for (**A**) drones and (**B**) workers. For both drones and workers, treatment groups without pollen showed significantly reduced body mass compared to Controls. *N. ceranae* infection alone did not significantly reduce body mass, however Malnutrition did. The boxplots show the inter-quartile range (box), the median (line within box), data range (horizontal black lines from box), and outliers (grey dots). Different letters indicate a significant difference between treatments.
